# “Get us partnerships!” - a qualitative study of Angolan and Mozambican health academics’ experiences with North/South partnerships

**DOI:** 10.1186/s12992-020-00562-7

**Published:** 2020-04-15

**Authors:** Isabel Craveiro, António Carvalho, Paulo Ferrinho

**Affiliations:** 1 Global Health and Tropical Medicine, GHTM, Instituto de Higiene e Medicina Tropical, IHMT, Universidade Nova de Lisboa, UNL, Rua da Junqueira 100, 1349-008 Lisbon, Portugal; 2 Centre for Social Studies, University of Coimbra, Praça Dom Dinis, 3000-104 Coimbra, Portugal

**Keywords:** Research partnerships, capacity building, Angolan and Mozambican health academics, Academic development, North/south collaboration

## Abstract

**Background:**

Sustainable Development Goal (SDG) 17 focuses on North/South partnerships for sustainable development. Literature on research partnerships and capacity -building often neglects how these processes are carried out in practice, their social impacts and participants’ subjective experiences.

Recognizing the increasingly global dimensions of Higher Education Institutions, the University Development and Innovation – Africa project (UDI-A) was designed to train lecturers and administrative staff of Angolan and Mozambican Universities through collaborations with European institutions, aiming at strengthening African academic and social landscapes through knowledge translation and dissemination.

This paper examines potential outcomes of UDI-A on participants’ academic pathways, investigating the conflict between different imaginaries of capacity-building and partnerships, focusing on how Angolan and Mozambican health sciences researchers experience international collaborations.

**Methods:**

Semi-structured interviews were conducted with seven health academics, as well as a focus group discussion involving all participants. These were recorded, fully transcribed, anonymized and coded to identify common themes. A consent form was signed by all participants.

**Results and discussion:**

UDI-A was considered innovative, fostering the improvement of pedagogical skills and increasing social entrepreneurship activities.

Participants arrived with a specific institutional mandate and believed that the training received should be incorporated into institutional practices to “modernize” these specific Portuguese speaking African Universities and the health sector. The institutional mechanisms put in place to attain this goal, Centres for Academic Development and Innovation (“CADIs”), were considered potential research and development hubs and drivers of academic and societal transformation.

Nevertheless, participants shared a sense of asymmetry (infrastructural, financial, in terms of access to information) between them and European trainers. Although this asymmetry was the underlying basis of this capacity-building project, they argued that UDI-A did not fully acknowledge their local contexts, compromising the prospective development of partnerships in the health field.

**Conclusions:**

More attention should be devoted to understanding how participants experience capacity building processes, integrating the diversity of their aspirations and perceptions into subsequent phases of the project, requiring the development of methodological innovations to increase the impact of these programs.

## Background

Sub-Saharan health care delivery systems share a number of challenges related to context (poverty, urbanisation, double disease burden), supply side bottlenecks, qualitative and quantitative deficit of human resources for health (HRH), commercialization of services and sub-optimal demand for the services offered [1] - Angola and Mozambique are no exception [2, 3]. These problems are partially addressed through development aid and North-South partnerships.

In recent years, development literature questioned the dichotomies between developing/industrialized and North/South [4]. Many of the buzzwords in development discourses are *“essentially contested concepts”* [5], combining “*general agreement on the abstract notion that they represent with endless disagreement about what they might mean in practice”* [6]. The meaning, scope and perceptions of partnerships are heterogeneous and up for debate.

Partnerships are at the heart of current discussions on aid effectiveness. There is a concern with ownership and accountability, as well as a normative discourse in favor of fair relations in international cooperation [7, 8].

The tensions that cross unequal power relations in partnerships result from unequal access to funding, knowledge and expert networks [8–10] - “*power and resource imbalances can lead to serious ethical challenges … including exploitation or “tokenism” within the partnership”* [11].

There is the need to develop research partnerships promoting a fair distribution of authorship and collaborative agendas that advance mutual interests while grounded in Southern priorities [12, 13]. The Sustainable Development Goal (SGD) 17 recognizes that research partnerships are key to attaining all the other SDGs, and over the past 20 years we have witnessed the development of recommendations and ethical guidelines on this topic, including the recently developed Research Fairness Initiative, a compliance tool that assesses how a specific institution “behaves” in partnerships [14].

Social science literature displays an ambivalent stance on research partnerships. On the one hand, it is recognized that partnerships may support technology transfer, capacity building and the improvement of academic and scientific systems in general. On the other hand, there are concerns with the appropriation of human and biological data, the relegation of scientists from the Global South to the status of mere field experts, not including southern partners as co-authors and not sharing the results with local communities. Research partnerships are often criticized for being a form of colonialism by other means [15], reproducing longstanding inequalities that characterize North/South collaborative projects and geopolitics. This has led to an increased concern with issues of power, access to funding and knowledge as well as to the intercultural and bureaucratic dimensions of partnerships, turning research partnerships into an object of sociological and anthropological enquiry [16], with strong impacts on public health policy [17, 18].

Health and research partnerships share many characteristics - health partnerships often include a research dimension and research partnerships usually go on to deliver service; common aspects include the value of multidisciplinary teams and the challenges of developing a shared understanding across national and cultural boundaries [19].

Collaborative approaches to address workforce development are on the increase [20]. The fragile capacity of African universities, shortages in HRH and weak leadership capacity are major concerns [21]. The Medical Education Partnership Initiative (MEPI) and the Nursing Education Partnership Initiative (NEPI) brought positive changes in health professions education [21]. Other examples are the Tropical Health and Education Trust (THET), the Primafamed network, the International Pharmaceutical Federation, the Global Pharmacy Education Development Network of UNESCO’s University Twinning and Networking Programme (UNESCO-UNITWIN) and the Training for Health Equity Network [22]. Meanwhile, partnership research – particularly health partnership research – is increasingly gaining track and visibility, stemming from the recognition that partnerships and capacity building are pivotal to addressing global health challenges, playing a crucial role in the enhancement of low and middle income countries (LMICs’) healthcare systems [19, 23, 24].

Literature on health research partnerships has increasingly relied on qualitative methodologies to monitor these international collaborations [25, 26]. However, little attention has been devoted to analyzing personal experiences within capacity building processes, overlooking the role played by narratives, aspirations and perceptions. Our article aims at addressing that gap.

## Methods

### Purpose

The aim of this paper is to examine partnerships and capacity building in practice, focusing on a small sample of Angolan and Mozambican Health Academics (AMHA) that took part in UDI-A, thus providing a qualitative contribution to existing literature on partnership research. The article is focused on participants’ expectations and perceptions of potential impacts of UDI-A on academic and professional pathways, as well as potential social impacts; we also explore heterogeneous imaginaries of capacity building and partnerships.

### Settings

UDI-A is coordinated by NOVA University of Lisbon (P1), involving partners in Angola and Mozambique - Universidade Agostinho Neto (P5)-, Universidade Katyavala Bwila (P7), Universidade Eduardo Mondlane (P6), Universidade do Lúrio (P8), and Europe - Kings College London (P2), Maastricht University (P4), Université Libre Bruxelles (P3). Angola and Mozambique are the two most populated Portuguese-speaking countries in Africa - P5 and P6 are the oldest, most prestigious and influential universities of their respective countries, while P7 and P8 are younger universities with great energy and activity aiming to become innovative forces in their own regions.

The preparation stage relied on consistent communication between partners belonging to Portuguese Speaking African Higher Education Institutions. The scientific and pedagogical areas considered priorities included economics and management, built environment and infrastructures, health sciences, humanities and social sciences. These fields were considered relevant to updating and improving student education, fostering sustainable and inclusive development in their regions; two crucial non-academic competences were also identified: student placement & entrepreneurship as well as international relations.

According to their areas of expertise, each one of the European (EU) partners assumed the coordination of the capacitation activities taking place in the different specific areas. Moreover, P4 has strong expertise in problem-based learning; P1, P2, P3 and P4 have several ongoing initiatives in the fields of social innovation and social entrepreneurship and all EU partners have excellent Offices of Student Placement, Entrepreneurship, and International Relations.

### Project structure

UDI-A started in late 2017. The aim of UDI-A is to improve the capacity of Higher Education Institutions (HEI) from selected African countries to better address local economic and social challenges, helping these institutions to foster sustainable and inclusive development through their academic and non-academic staff trained during the project.

UDI-A has four specific objectives:

- To improve the quality of education, research and service of African Institutions by updating knowledge and skills of staff, promoting interdisciplinary approaches to research and education.
- To bring Universities closer to local societies, engaging relevant stakeholders in activities involving both students and staff.
- To support the internationalization of African Partners by promoting the international mobility of staff and students.
- To promote a culture of social innovation and social entrepreneurship.

In order to reach these objectives, UDI-A put in place an International Capacitation Programme (ICP) focusing on four scientific areas identified based on a participatory approach by African UDI-A partners: economics and management; built environment and infrastructures; health sciences; humanities and social sciences. These areas are crucial to allow HEIs to contribute to sustainable and inclusive development at the regional level.

With support from their HEI, motivated African academic and non-academic staff (Champions) and students (Junior Champions) will – after updating technical and scientific skills through formal training, self-study and non-formal learning initiatives - connect with local corporations and relevant social actors, ideally fostering social and institutional change.

UDI-A’s impact will be twofold: first, African partners will count on a team of Champions with a strategic impact on their Institutions; second, there are no institutional mechanisms currently in place to foster sustainable and inclusive development, and UDI-A will lead to the creation of Centres for Academic Development and Innovation (CADIs), focused on Academic Development and Innovation. UDI-A recognizes that capacity building involves not only individual training but also broader institutional changes; therefore, CADIs encompass organizational reconfigurations at the institutional level; changes in physical infrastructures (new buildings and equipment); a multiannual strategy and corresponding budget and the engagement of stakeholders [27].

### Selection of champions

Ten Champions (8 academics of the four targeted scientific areas, 2 non-academics) were selected in a first phase of the project by each African institution and participated in an ICP to update technical and scientific skills through formal training, self-study and non-formal learning initiatives. In the first phase, Champions travelled to Europe, where they were distributed among the different European partners (depending on their scientific areas of expertise), undergoing training in their respective areas. In the subsequent phases of the capacity building program, European partners went to Portuguese Speaking African Universities to provide training in various scientific and pedagogical fields.

For this study, all health sciences Champions were purposively sampled based on their particular experience on health HEI (Table 1). The institutional representation of the participants is as follows: four from Mozambique (2 from Lúrio University and 2 from Eduardo Mondlane University) and three from Angola (2 from Katyavala Bwila University and 1 from Agostinho Neto University).

**Table 1:** Participants’ profile – health sciences Champions

Participants	Age	Research area
Mozambique	26	Optometry
	27	Pharmaceutical sciences
	39	Biotechnology
	38	Chemistry
Angola	24	Medicine
	26	Physiology
	41	Biology and genetics

### Study design

We developed a qualitative study on UDI-Africa and our analysis focused exclusively on the Champions from the health arena, relying on semi structured interviews and focus group discussions.

### Data collection

As our study focuses on examining partnerships and capacity building in practice, we resorted to qualitative research methods, recognizing the potential of qualitative approaches to allow participants to reflect on their individual and collective experiences throughout these collaborative processes [28]. Semi-structured interviews were conducted between March–April 2018 with seven AMHA, as well as a focus group discussion involving all participants. The data collection took place in Lisbon during the first phase of the UDI-A project, when Champions travelled to Europe. Although this is a qualitative study, it is still a relatively small number of interviews, but they comprise all AMHA involved in the ICP. A flexible interview schedule was prepared, focusing on: expectations about UDI-A’s impact on academic and professional pathways; potential societal impacts; discourses on capacity-building and partnerships. The focus group drew on some of the topics shared during the interviews and allowed the whole group of AMHA to collectively reflect and identify their main experiences and perceptions, and was key to supporting our data analysis, but the semi-structured interviews were crucial to exploring individual perspectives.

The interviews and focus group were audio recorded.

### Data analysis

The interviews and focus group were fully transcribed, anonymized and coded to identify common themes, with both inductive and deductive coding, and were informed by a template approach [29]. A provisional template was created with a set of deductive themes that were broad and relevant to the study questions and associated literature. Two researchers (IC and AC) independently read the transcripts, applied the template to a subset of the data and discussed the coding scheme and emerging themes. A revised template was then applied to all transcripts. As coding proceeded, additional themes emerged.

## Results

Key results include four themes: R&D asymmetries between Europe and Africa; partnership expectations; failed expectations; positive impacts.

### R&D asymmetries between Europe and Africa

The first theme refers to the asymmetry between R&D in Europe and Africa, reinforcing the relevance of UDI-A and justifying AMHA’s interest in the project. According to a Champion from Angola:

“*The differences in terms of R&D between* European Institutions *and our institutions are abysmal...they can’t even be compared, but we are open to learning … and taking the first steps … We have R&D, but not as … comprehensive as yours.*” (C1)

He continued to delve into this topic and provided a description of some of Angola’s structural problems:

*“They [European Institutions] already have a structure … focused on scientific research … there are offices supporting research … what we have is an embryo … we don’t have an institutional effort towards scientific research …*” (C1)

Mozambican and Angolan academics shared the same concern - the financial context is dire, undermining R&D:

“*… our human resources are well trained and knowledgeable. But there is no equipment …*” *(*C2)

“*… we don’t have the equipment, we don’t have sufficient libraries, it’s very hard to do research … Infrastructures, equipment … affect everything, and we lack them.”* (C3)

While recollecting his experience in the Netherlands, a participant emphasized how the overall conditions were so much better there:

*“Laboratories are well equipped, libraries are equipped. Mediatheques, everything! We visited a library and sincerely we almost got lost there!”* (C3)

One aspect mentioned by another Angolan researcher was research dissemination - this problem affects African R&D [30], and the interviewee highlighted the gap between research and publishing:

“*In Africa there is research, but often it doesn’t get published. When there are conferences, researchers … present their work, but only those attending have access to that information … if I want to launch a research project, I’m clueless … that topic could even be developed by someone else in my country!”* (C5)

Since research dissemination is undeveloped, researchers from the Global North frequently visit Angola to obtain empirical data, publishing articles without crediting their Angolan counterparts, a claim supported by current literature [31]:

*“There is plenty of international research on a plant that is known to … cure gastric ulcers. That plant … only exists in Angola. That article was published in an international journal. But the Angolan researcher is only briefly mentioned …*” (C5)

A champion from Mozambique highlighted the differences between Africa and Europe regarding research dissemination - according to him, something should change:

“*There are lecturers that haven’t published in ten years! I came here and I asked how many articles they published every year … it was more than a hundred! Our department doesn’t even publish 10 … For a forty-year old University! It’s really bad …*” (C6)

Another Champion from Mozambique highlighted how the lack of a strong research structure in his University limited the reach of its research:

*“Many studies are only disseminated inside the Institution … not reaching our city or the world. Therefore, research … starts and dies right away … …*” *(*C7)

### Partnership expectations

AMHA were interested in improving pedagogical and research skills, and had personal expectations and an institutional mandate to enhance their Universities R&D profile through partnerships. According to a Champion from Angola:

“*The main motivation to join UDI-A was to create a Center for Scientific Research … the benefits generated by UDI-A will stem from partnerships with researchers in Europe. It will allow us to develop projects, to become aware of how they are funded and so on.”* (C1)

These partnerships would rely on the mobility of academics, having a positive impact in Africa:

“*The main role of … partnerships, from an institutional point of view, is to promote mobility ( …*) *to develop skills and improve human resources. And also opportunities to develop research projects.*” (C1)

According to a Champion from Mozambique, initial expectations included practical aspects:

*“I wanted to know how to manage a course. How to … increase academic performance and … carry out intervention projects. Social and community intervention ( …*). *… I also wanted to obtain experiences from other countries on these subjects, adapting them to our reality.*” (C2)

This participant highlighted that she had an institutional mandate - her stay in Europe should benefit her institution:

*“My Institution has expectations that we will return with ideas of how to manage certain courses, how to help the University create that research center [CADI]. Its development will lead to new ideas and innovation, at the institutional and academic levels. ( …*) *They expect changes when we return …*” (C2)

According to another Champion, UDI-A would generate stronger partnerships, increasing the social impact of research:

*“In Angola we already have the center for educational sciences … we want to join them to make sense of all the information we’re getting here, to develop health research with a higher social impact …*” (C3)

A Champion from Angola mentioned that partnerships, social entrepreneurship and pedagogical innovations were the main drivers to join the project:

*“Some things that drew my attention were social entrepreneurship and pedagogical innovations. What we would learn from others – how to innovate, how to transform, and also partnerships with relevant institutions or individuals … When we return they will ask us if we established partnerships …*” (C5)

The same academic reiterated that the main goal was *“To create a CADI in each institution taking part in the project”* (C5), reinforcing the institutional impact of UDI-A.

A Champion from Mozambique applied to join the project because it coupled pedagogical and research aspects:

“*What caught my attention were two aspects: pedagogical and scientific capacity building, … my two fields of work. That’s why I applied*.” (C7)

However, his main motivation was to improve research skills, as Mozambican R&D is still underdeveloped.

### Failed expectations

AMHA argued that UDI-A was overly focused on social entrepreneurship, soft skills and pedagogy instead of fostering research skills, leading to some negative feedback.

According to a Champion from Angola, UDI-A was not sufficiently focused on research skills - he felt that his motivation letter was disregarded:

*“In my motivation letter I mentioned that I was interested in social entrepreneurship and scientific research. However, I’m realizing the program has been changed … they included activities which were not planned …*. *We had … field visits … in the last days of our stay … we were expecting … … more time allocated to scientific research.” (*C1)

According to a Champion from Mozambique, this could limit UDI-A’ impact:

*“We had higher expectations regarding the health sector, we wanted something … more developed. There was a big disconnect* [between expectations and experiences] … *… Pedagogical aspects were positive, but in terms of the health field my gains are very reduced …*. *I believe this will have a negative impact …*.” (C2)

This disconnect illustrated an asymmetry between trainers and Champions. According to an AHA from Angola, this was particularly worrying due to her institutional mandate:

*“Here we do not feel listened to … we just receive information … it’s frustrating … we ask ourselves: … “everything I learned today, how am I going to apply this to my context?”. …*. *What my University expects is the establishment of partnerships … and so far I have nothing …*. *Even when it comes to social entrepreneurship … everything is too vague …*” (C4)

A Champion mentioned that he expected UDI-A to focus on research skills, criticizing the emphasis on social entrepreneurship disconnected from local contexts:

*“Initially I thought that UDI-A would allow us to overcome our research gap … I’m getting slightly disappointed. Regarding social entrepreneurship … if it’s a reality whose context we don’t know then what is the point? You need to take local aspects into account … If you want to solve a social problem, you need to be in that community … Social initiatives fail because people don’t understand what is happening there …*” (C7)

The focus group discussion was a great opportunity to explore these issues, reinforcing some of the ideas that arose during the interviews. A Champion mentioned that UDI-A should include capacity building in research methods:

*“The scientific component we are developing through occasional partnerships and individually … it’s not in the program”* (C7)

Overall, the main concern was returning to Africa without formal partnerships:

*“Our managers there (in Angola), when they sent us here they told us: “look, get us partnerships!”. And we’re returning with empty hands. What they wanted was someone saying “look, we’re gonna help!”. With money, information, consulting … something concrete!”* (C5)

### Positive impacts

AMHA recognized that they acquired relevant skills in social entrepreneurship, teaching and research, and they found PBL (Problem-Based-Learning) helpful to improve their pedagogical skills. A Champion summarized UDI-A’s positive impacts:

*“The experience was positive …*. *PBL was very useful. Certainly we will use it in our countries … entrepreneurship … was very useful …*” (C2)

Visits to research centers allowed AMHA to interact with European researchers:

*“We carried out field visits according to our research interests. We visited research centers …*. *Some of them were willing to support our projects, attending our congresses, workshops … And there was the possibility of organizing joint academic conferences …*” (C1)

A Champion from Mozambique was enthusiastic about UDI-A, dwelling into the personal and institutional significance of partnerships:

“*… those partnerships will make a difference, we will have contacts with researchers in … community and public health …*. *From now on, any project … in my department … will be able to recognize that: “look, those in Portugal, in Lisbon, are working on this …*” *.* (C2)

Field visits were praised, allowing AMHA to contact European counterparts, providing a hands-on approach to research:

“*We visited research labs at the Medical Sciences Faculty. We met various researchers …*. *to understand how things work and how they are being developed. We gained some “know-how” about what they are doing, something we need urgently … we suffer from an R&D gap …*” (C3)

When asked about partnerships, this participant argued for the need to develop collaborative projects between European and African researchers. This possibility was reiterated by another Champion, who mentioned that this was her first chance to travel outside Mozambique:

*“The opportunity to visit different labs … it opens up our minds! Great things are happening! There are people who can help us … it’s my first chance of travelling and appreciating how things are happening outside my country … I could only resort to articles or books …*” (C4)

A Champion from Angola mentioned some of the partnerships focusing on pedagogical methods established with Maastricht University that could be implemented in Africa:

*“We established partnerships with people available to collaborate remotely ... Teaching us how to do it, giving us feedback, providing online support.”* (C5)

Nevertheless, Champions recognized that Portuguese Speaking African institutions would need to accommodate their new experiences, which could be problematic. As put by an Angolan participant:

*“The integration of our experiences must be progressive … it will imply a restructuring of the Faculty. … Facilities are not ready. They are prepared for the traditional method, the resources, libraries, … that needs to be adjusted … we also need to change people’s minds.”* (C6)

Although AHA recognized that the ICP was beneficial, there was also some criticism.

A quality assurance report was applied in order to assess participants’ evaluation of the ICP through two online surveys. This involved Champions from all research fields and not exclusively Angolan and Mozambican Health Academics. Some of the positive aspects mentioned included opportunities for networking with African colleagues and the fact that the scientific topic training period was useful for the progress of personal development plans. However, only 73% respondents agreed that the scientific topic training period achieved the learning objectives set at the beginning, and the issue of how to identify the type of stakeholders to involve in local projects also raised some concerns. Our paper is exclusively focused on the qualitative protocol undertaken to analyze AMHA’s perceptions of the ICP, although some of the aspects shared by our sample confirm some of the previously mentioned concerns [32, 33].

## Discussion

Health Champions recognized the existence of an R&D gap in Africa, and their main motivation was developing research skills and partnerships with European institutions, leading to the development of CADIs.

Although UDI-A aimed at reducing R&D unbalances, participants felt the process was asymmetrical, complaining about long lectures on social entrepreneurship and soft skills, resembling what Paulo Freire referred to as “banking education” [34]. This is common to capacity-building projects, and partnerships should be based on the recognition that partners often have different backgrounds, needs and expectations [35]. This requires interactive approaches doing justice to local, personal and institutional aspirations, as unbalanced power relations are a recurrent cross-cutting issue while analyzing partnerships.

There was also a clash between capacity building and research partnerships. Participants had strong expectations regarding research partnerships: however, UDI-A focused on pedagogical aspects and social entrepreneurship. Although AMHA recognized that these would improve their academic performance, the focus on capacity-building did not meet their motivations and undermined the potential to establish formal partnerships.

The capacity-building program focused on soft skills, social entrepreneurship and individual capacities, recognizing the potential of Champions as agents of institutional and social change. However, UDI-A was criticized for not taking into account local contexts, reproducing some of pitfalls attributed to partnership programs [12] - setting priories without integrating local stakeholders’ inputs [36] - reinforcing the contested nature of “partnerships” and “capacity-building”.

Our data suggest that methodological innovations [20] must be mobilized to attend to the expectations and cultural specificities of participants. The Community of Portuguese Language Countries (CPLP) has also been prolific in the development of methodologies for learning and social action [14]. Moreover, within evaluation research there is a trend towards intercultural approaches recognizing that indicators are culture-specific [37], favoring the interactive and participatory development of the variables underlying those indicators [38].

Champions recognized UDI-A’s individual, institutional and social potential. However, their expectations were not fully met; a more balanced methodology could have produced stronger results, namely the systematic establishment of research partnerships.

As our protocol was focused exclusively on the health field, drawing on a relatively small sample, our findings shouldn’t be generalized to participants from other academic fields who took part in the ICP, but as we saw in this paper, quality assurance reports were developed within UDI-A, giving Champions (from all fields) the chance to evaluate the ICP. Although there are some overlapping topics, our findings are specific to AMHA, since we considered UDI-A a unique opportunity to understand the narratives of Angolan and Mozambican global health agents as they were participating in a partnership-based training / capacitation process.

We aim to extend our research protocol to subsequent phases of UDI-A, including the perspectives of other participants in the ICP, and future quality assurance reports will also allow us to assess whether AMHA’s experiences can be generalized or if they are specific to this group.

## Conclusions

There are clear discrepancies between the adopted project methodologies and the initial expectations of AMHA, as well as unmet challenges to integrate both perspectives. UDI-A is an ongoing project, involving a process of mutual learning – there is the potential of building fair partnerships but AMHA’s aspirations must be integrated throughout all stages. Therefore, our analysis provides critical notes and insights that may be useful in the development of future capacity building programs, namely those involving Higher Education Institutions and AMHA facing health challenges that require the rapid improvement and responses of LMICs’ healthcare and research systems.

While recognizing that our sample is relatively small, although it includes all health academics, the experiences and perceptions presented in this article highlight some of the setbacks and limitations often attributed to North/South partnerships, indicating the need of developing collaborative methodologies that attend to the expectations, aspirations and visions of participants from the Global South. Moreover, and in line with current concerns with equitable partnerships, we aim at including Champions as co-authors in future publications stemming from UDI-A.

## Data Availability

The dataset analyzed during the study is available from the corresponding author on reasonable request.

## Abbreviations

AMHA: Angolan and Mozambican Health Academics
CADI: Centre for Academic Development and Innovation
HEI: Higher Education Institution
HEPI: Health-worker Education Partnership Initiative
HRH: Human Resources for Health
ICP: International Capacitation Programme
NEPI: Nursing Education Partnership Initiative
PBL: Problem Based Learning
R&D: Research and Development
THET: Tropical Health and Education Trust
UDI-A: University Development and Innovation – Africa
